# Characterize and Address Mental health Problems in University Students (CAMPUS Study): preliminary results

**DOI:** 10.1192/j.eurpsy.2024.1133

**Published:** 2024-08-27

**Authors:** M. Nosè, G. Muriago, F. Tedeschi, G. Turrini, C. Barbui

**Affiliations:** Department of Neuroscience, Biomedicine and Movement Sciences, Section of Psychiatry, University of Verona, Verona, Italy

## Abstract

**Introduction:**

The transition phase from late adolescence to early adulthood, which corresponds with the period of university life, is a time that offers important opportunities for personal growth. However, this developmental phase also concurs with the peak period of risk for the onset of mental health disorders. For this reason, the literature clearly identifies university students as a vulnerable population group for psychogical distress and mental problems. Digital psychological interventions and e-mental health solutions are emerging as a promising solution for university students, particularly appealing due to their anonymity, portability and ease of access. Hence, the World Health Organisation has developed several psychosocial e-mental health tools including Doing What Matters in Times of Stress (DWM), which has been consistently shown effective in various vulnerable populations. These data provide the framework for the CAMPUS study that is intended for students attending the University of Verona.

**Objectives:**

The main objective of this project is to adapt the WHO psychological intervention called “Doing What Matters in Times of Stress” (DWM) to this target population and to evaluate the effectiveness, feasibility, and acceptability of WHO’s DWM as a psychological strategy for effective mental health prevention and promotion, and for reducing psychological symptoms and distress in university students. Secondary objectives of the project include to evaluate the fidelity of DWM, to assess factors associated with its implementation and effectiveness and to co-create the necessary local conditions for implementation and up-scaling of DWM.

**Methods:**

The CAMPUS study is a prospective non-randomized follow-up study. The target population is composed by university students of University of Verona, Italy. The online assessments, which are collected pre and post intervention, consist of an ad-hoc sociodemographic information page, and four self-administered questionnaires assessing psychological distress, depression and anxiety symptoms, and psychological well-being. In addition, implementation checklists will be administered to assess the acceptability, appropriateness and feasibility of the intervention.

**Results:**

Preliminary results on a sample of 300 students attending University of Verona show that the adapted DWM intervention promote students’ psychological well-being and reduce the level of psychological distress as well as the risk for the later development of a psychopathology. Moreover we expect that future results would include data on the effectiveness, feasibility, and acceptability of the adapted DWM intervention among university students

**Conclusions:**

These results provide valuable information for mental health promotion and support programs for university students, as well as insights into factors influencing its implementation and suggestions for future scaling of the intervention.

**Disclosure of Interest:**

None Declared

